# Palliative care for patients with Parkinson’s disease: study protocol for a mixed methods study

**DOI:** 10.1186/s12904-017-0248-2

**Published:** 2017-11-25

**Authors:** Herma Lennaerts, Marieke Groot, Maxime Steppe, Jenny T. van der Steen, Marieke Van den Brand, Dorian van Amelsvoort, Kris Vissers, Marten Munneke, Bastiaan R. Bloem

**Affiliations:** 1 Radboud university medical center, Nijmegen, 6500 AB The Netherlands; 2 Department of Neurology, Nijmegen, 6500 AB The Netherlands; 3 Department of Anaesthesiology, Pain and Palliative Care, Nijmegen, 6500 AB The Netherlands; 4 Department of Primary and Community Care, Nijmegen, 6500 AB The Netherlands; 5 Leiden University Medical Center, Department of Public Health and Primary Care, Leiden, The Netherlands; 6 Caregiver, Dutch Parkinson Association, Bunnik, The Netherlands

**Keywords:** Parkinson’s disease, Palliative care, Patient experiences, Mixed methods, Study design

## Abstract

**Background:**

Parkinson’s disease (PD) is a chronic, progressive neurological disorder with many intractable consequences for patients and their family caregivers. Little is known about the possibilities that palliative care could offer to patients and their proxies. Guidelines strongly recommend palliative care to improve the quality of life and – if needed – the quality of dying. However, providing palliative care to persons with PD involves specific challenges. For example, a timely initiation of palliative interventions is difficult because due to the gradually progressive nature of PD, there is often no clear marker for the transition from curative towards palliative care. Furthermore, there is little evidence to indicate which palliative care interventions are effective. Here, we describe the contours of a study that aims to examine the experiences of patients, (bereaved) family caregivers and professionals, with the aim of improving our knowledge about palliative care needs in PD.

**Methods/design:**

We will perform a mixed methods study to evaluate the experiences of patients, (bereaved) family caregivers and palliative care professionals. In this study, we focus on Quality of Life, Quality of Care, perceived symptoms, caregiver burden and collaboration between professionals. In phase 1, we will retrospectively explore the views of bereaved family caregivers and professionals by conducting individual interviews and focus group interviews. In phase 2, 5–15 patients with PD and their family caregiver will be followed prospectively for 8–12 months. Data collection will involve semi-structured interviews and questionnaires at three consecutive contact moments. Qualitative data will be audio recorded, transcribed and analyzed using CAQDAS. If patients pass away during the study period, a bereavement interview will be done with the closest family caregiver.

**Discussion:**

This study will offer a broad perspective on palliative care, and the results can be used to inform a palliative care protocol for patients with PD. By describing the experiences of patients, (bereaved) family caregivers and professionals with palliative care, this investigation will also establish an important ground for future intervention research.

## Background

Parkinson’s disease (PD) is a common neurodegenerative disorder, affecting approximately 1% of the population over the age of 65 in Western countries [1]. PD is an incurable disease, and treatment exists of symptomatic strategies (such as suppressing symptoms, or offering support). As PD progresses, patients can experience a wide range of symptoms like immobility, pain, fatigue, sleeping problems, cognitive deficits and dementia [2–4]. A nursing home admission is inevitable in 20–40% of PD patients [5–9]. Disease management in advanced stages of PD becomes increasingly difficult, whereas the emphasis on quality of life becomes more important. Patients with PD experience considerable discomfort at the end of life; in fact, their symptom burden is comparable to that of advanced cancer patients [2, 10].

Palliative care is “an approach that improves the quality-of-life of patients and their families facing problems associated with life-threatening illness, through the prevention and relief of suffering by means of early identification and impeccable assessment and treatment of pain and other problems, physical, psychosocial and spiritual” [11]. However, there is barely any knowledge about the effective and useful components for palliative care in PD. [12] Hasson et al. reviewed the current evidence and reported several challenges that may occur in clinical practice. Problematic is the (timely) identification of PD patients and their palliative care needs. The absence of a concrete starting point for focusing on palliative care needs makes it difficult to identify palliative care needs during the disease course.

A few specific disease symptoms have been recognized as indicators for palliative care needs. This includes the first episode of aspiration and the occurrence of key clinical features such as visual hallucinations, regular falls, dementia and admission to residential care; patients experiencing such symptoms might need special attention and start of the palliative care phase can be considered [12, 13]. However, the appearance of these specific symptoms do not always lead to recognition of palliative care needs or referral to palliative care services.

PD patients and family caregivers described a lack of knowledge about palliative care services [14]. And if they had received care from a palliative care service, then coordination of care was reported to be poor [14–16]. Studies conclude that family caregivers often feel alone in the care for the patient with advanced PD [14]. Only few PD patients and family caregivers receive palliative care in contrast to patients with malignant diseases [17].

Most studies evaluated patients with PD needs in early stages and paid less attention to patients with advanced PD [18, 19]. However, a few studies found unmet needs in advanced PD [20] Patients with advanced PD are cared for by family members, usually older spouses [21]. Schrag et al. reported that for patients at advanced stages, caregiver burden rises with increasing disability, and also with the appearance of symptoms such as hallucinations, depression and falls [22]. Family caregivers of patients with advanced PD reported considerable changes in their life [14, 15, 20, 21, 23]. More specifically, spouses felt that their primary role in the relationship changed, due to cognitive deficits and speech problems of the patients. Family caregivers struggled with caring for their loved ones as long as possible and specifically with the point of admission to a nursing home. Studies also reported that family caregivers had fewer social contacts and opportunities to socialize, had a reduced financial income and tended to experience poorer health; this is particularly related to features of late stage PD [21, 22]. Likewise Carter et al. explored pre-death grief in family caregivers of advanced PD patients and the results suggests that pre-death grief was a significant finding in family caregivers and was more associated with the presence of patient’s cognitive decline [24].

Four studies reported professionals’ experiences with (the concept of) palliative care and PD [25–28]. Waldron et al. showed that professionals often misconceive the putative value of rehabilitation in the palliative care phase. Moreover, professionals felt unsure about the care they delivered in this phase. Several studies reported that professionals experienced a lack of education and competence in this field. Furthermore, the collaboration between palliative care services and more general professionals was missed. Perceived barriers in the collaboration were inadequate referral and lack of communication.

In order to address the existing knowledge gap, we will now launch the ParkinsonSupport study, with the aim of identifying the experiences with end of life issues among both people with PD, their family caregivers and the professionals involved. Part of this project is a qualitative after-death study with professionals and beraeved family caregivers, as well as a prospective multiple case study with patients and their family caregivers.

These issues will be explored by asking patients, (bereaved) family caregivers and professionals about their experiences and ideas about optimizing palliative care. Important aspects of this exploration also include identifying terminal care strategies that have already been used in practice by professionals, and the identification of ‘red flags’ in order to get more insight information to start palliative care interventions timely. Additional questions emerging from our main research question include: [1] What opinions do patients, (bereaved) family caregivers and professionals have about the quality of care provided? [2] What are specific disease symptoms that may assist in the timely identification of palliative care needs in patients with advanced PD? [3] How do disease care needs progress over 8-12 months in PD patients and family caregivers? [4] What are the major decisions and symptoms in dying with Parkinson disease?

The objective of this paper is to provide a detailed description of the study design and research protocol.

## Methods/study design

This study includes two parts. We will start with an explorative, qualitative after-death study. In-depth individual and focus group interviews with bereaved family caregivers and professionals involved in the care for people with PD will be held. The second part consists of a qualitative prospective multiple case study design [29]. This design facilities the understanding of circumstances and experiences of patients with advanced PD and their family caregivers.

### Explorative after-death study

A topic list will be developed based on a literature study. A pre-test will be held in a test panel to ensure comprehensibility and completeness of the topic guide. Subsequently, 10 professionals coming from different disciplines and 10 bereaved family caregivers will be interviewed. Also, focus group interviews will be held with professionals until saturation is reached. This design allows for obtaining in-depth information about the needs of bereaved family caregivers of patients with advanced PD and professionals.

### Multiple-case study

Patients and their family caregivers will be involved in the multiple-case study. We will follow around 5 to 15 PD patients (and if present one family caregiver per patient) to address how quality of life and quality of care is being perceived and any changes over 8 to 12 months. PD patients will be included based on an affirmative answer from the attending physician on the so-called “surprise question”: ‘Would I be surprised if this patient died in the next 12 months?’ [30]. Patients will be visited at home at baseline, at 6 months and at 12 months after baseline to assess experiences, symptoms, quality of life and quality of care. Data will be collected by in-depth interviews with the PD patient as well as his/her family caregiver. Alongside the interviews, questionnaires and documents (e.g. medical records) will be used. If patients die during the study period, the closest family caregiver will be invited for a bereavement interview.

#### Selection of participants

We will recruit professionals via the Dutch ParkinsonNet, a professional Parkinson-specific healthcare network with full nationwide coverage, consisting of 69 regional networks with more than 3000 professionals specialized in the treatment of patients with PD, including groups of specifically trained neurologists, PD nurse specialists and nursing home physicians [31, 32]. We also include bereaved family caregivers via a partnership with the Dutch Parkinson Patient Association, and via the web-based community for PD patients and families (www.parkinsonconnect.nl). For the case study, we will identify patients in advanced stages in hospitals, general practices and nursing homes. We will ask neurologists, PD nurse specialists and elderly care physicians who are member of ParkinsonNet to screen their population. Signed informed consent forms from the patient and family caregiver will be obtained before study entry.

### Explorative after death study

Bereaved family caregivers who have been involved in the care for a patient with advanced PD during the past two years, will be invited for an individual interview. We aim to conduct 10 individual interviews. We also aim for a diversity of professional disciplines in the interviews, as the care for PD is multi-professional. Focus groups will be heterogeneous. Participants need to meet the inclusion criteria (see “Inclusion criteria for bereaved family caregivers and professionals”) in order to be eligible for participation.

#### Inclusion criteria bereaved family caregivers

1. 18 years or above;
2. cognitively able to participate in interviews;
3. bereaved family caregiver who has been involved in the care for a patient with advanced PD over the last 2 years.

#### Inclusion criteria professionals

1. Professionals who were involved in the care for a patient with advanced PD over the last 2 years.

### Multiple-case study

We aim to achieve a total sample of around 5–15 patients and family caregivers. Patients (and if present one family caregiver per patient) will be recruited by their attending neurologist, PD nurse specialist or general practitioner. A family caregiver, defined as the person who non-professionally takes care and supports the patient for most of the time, will be identified by the patient. Family caregivers need not necessarily be a family member. Participants need to meet the inclusion criteria (see “Inclusion criteria for patients and family caregivers”) in order to be eligible to take part in the study. In order to gain variation in patients’ experiences with care provision, we strive to purposively recruit patients who live at different settings e.g. at home or in a nursing home.

#### Inclusion criteria patients

1. 18 years or above;
2. Diagnosis of “idiopathic PD” according to UK Parkinson’s Disease Society Brain Bank clinical diagnostic criteria;
3. Patients who are suffering from late-stage Parkinsonism classified according to Hoehn and Yahr stage (H&Y) IV or V in the “On”-state; OR who have developed significant disability (Schwab and England stage 50% or less) in the “On”-state;
4. Cognitively able to complete questionnaires and to participate in interviews;
5. The patient’s attending doctor answers “No” to the surprise question: “Would you be surprised if the patient died within the coming 12 months?”

#### Inclusion criteria family caregivers

1. 18 years or above;
2. Cognitively able to complete questionnaires and to participate in interviews;
3. Identified by the patient as family caregiver.

### Data collection

#### Explorative after death study

We will start with semi-structured interviews with 10 bereaved family caregivers and 10 professionals. We will use the COREQ-checklist for reporting qualitative studies as far as this is applicable to our study. Based on the literature, a semi-structured interview guide will be developed for the individual interviews (see Table 1 for topic areas). Individual interviews will be conducted by trained researchers from the project team. All interviews will be audio recorded and transcribed verbatim. Two researchers undertake the data collection independently and roughly analyze the transcripts. Focus group interviews will be used to obtain further insight into the experiences of the professionals with (their) palliative care, until saturation is reached. Common and consistent themes within individual and focus group interviews will be drawn together in the final content analysis.

**Table 1:** Interview topic areas for individual interviews

Bereaved family caregiver	Professionals
Demographic data: age, gender, years of caring	Demographic data: age, gender, profession, years of experience, highest level of education
- History of family member’s illness - Exploration of problems and needs of the patient during advanced PD and end of life phase (palliatieve care domains: physical, psychosocial, mental and spiritual) - Exploration of problems and unmet and met needs of the bereaved family caregiver (palliative care domains: physical, psychosocial and spiritual) - Problems and needs in the dying phase (including end of life decisions) - Exploration of the collaboration between professionals and access to palliative care services - Bereavement support	- Defining palliative care - Perceptions of patients’ and carers’ palliative care needs - Barriers and facilitators in providing palliative care - Exploration of ethical issues and end of life decisions - Exploration of the collaboration between professionals and palliative care services - Expectations and/or future improvements

#### Multiple-case study

The multiple case study consists of three consecutive contact moments with patients and family caregivers with an interval of approximately 6 months (baseline, month 6, month 12). During a home visit, we will conduct semi-structured interviews and complete questionnaires. We will use a questionnaire with Parkinson-specific supplements; the Edmonton Symptom Assessment System Scale modified for Parkinson disease (ESAS-PD) [33]. Socio-demographic data; age, gender, marital status, education, employment status, and relationship between patient and family caregiver, will be collected at baseline.

At each contact moment, we will also separately interview the family caregiver at home. Family caregivers may feel uncomfortable in the precense of their partner to speak about the impact of advanced PD, patients’ mental health status or caregiver burden. The data collection methods that will be applied to assess the outcome parameters are described in Table 2 for the case study. If a patient dies during the study period, a bereavement interview will be done with the family caregiver.

**Table 2:** Data collection schedule

Outcome parameter	Data collection method	T0	T1	T2
Patient				
Disease severity, perceived symptoms	ESAS-PD	X	X	X
	Hoehn & Yahr, Schwab & England	X	X	X
Functional Assessment	FACT-G	X	X	X
Experiences with palliative care, Quality of Care	Semi-structured interview	X	X	X
Quality of Life	PDQ 8	X	X	X
Family caregiver				
Experiences with palliative care, Quality of Care	Semi-structured interview	X	X	X
Perceived symptoms of patient	ESAS-PD	X	X	X
Caregiver Burden	ZBI	X	X	X

We will ask patients if they want the presence of a family caregiver during the interview. Patients with PD in an advanced stage are vulnerable and may have speaking problems. Therefore, we expect that patients want to be supported by their family caregiver. If this is the case, we will, in order to minimize influences of opinions between patients and family caregivers, emphasize the role of translator for the family caregiver in the interview with the patient.

### Questionnaires

Questionnaires were selected based on: validity and/or applicability in patients with PD and/or palliative care, time needed for completion, and available translations into Dutch. Patient’s disease severity and the ability of independent living will be measured with Hoehn and Yahr (H&Y) scale and Schwab & England [34, 35].

#### Edmonton symptom assessment scale Parkinson disease (ESAS-PD)

The Edmonton Symptom Assessment Scale Parkinson Disease will be filled in by patients and family caregivers to measure perceived disease symptoms of patients [33]. It is tested but limitedly used in palliative care for patients with Parkinson Disease in contrast to the original ESAS which is widely used, tested and validated. The completion time is short, approximately 7 min and the ESAS-PD is available in Dutch [36].

#### Zarit burden interview (ZBI)

The Zarit burden Interview will be used with family caregivers to measure their perceived burden [37]. The ZBI is widely used and extensively tested, also in studies with PD patient caregivers. It has been translated into Dutch and has a completion time of approximately 10 min.

#### Parkinson disease questionnaire 8 (PDQ-8)

The Parkinson’s Disease Questionnaire 8 is a disease-specific instrument that captures PD patients’ perception of their illness. The PDQ-8 covers eight dimensions of ill-health, and contains 8 questions. PDQ-8 is a shorter version of the PDQ-39, which is widely tested and validated. The PDQ-8 has been used in varies studies with good psychometric results [38–40].

#### Functional assessment of cancer therapy – General (FACT-G version 4)

FACT-G will be used to assess the patients’ functional status. The instrument is used and validated in mainly patients with cancer and a small group of patients with chronic diseases. The FACT-G is a self rating questionnaire, which contains questions about the patient’s own ability of functioning at four subscales (physical wellbeing, social wellbeing, emotional wellbeing and functional wellbeing) [41]. It has been translated into Dutch and has a completion time of approximately 5-10 min.

The expected duration of the entire study period will be 24 months, including recruitment, data collection and analysis. Data collection has started in September 2016 and will finish in September 2018.

### Data management and analysis

We will analyze data from individual interviews and focus group interviews using a thematic content analysis approach. Individual- and focus group interviews will be tape recorded and transcribed verbatim. Two researchers will collect the data and will independently analyze transcripts and build a codebook. The codebook allows for comparison and contrasting between interpretations, helping to enhance inter-rater reliability. Common and consistent themes will be drawn together in the analysis. Transcriptions will be analyzed using content analysis techniques supported by a qualitative analysis software package (CAQDAS).

Interviews will be analyzed in cases and by means of the same iterative procedure as described above. During this analytical phase, we will integrate the quantitative variables and qualitative findings so as to draw a more complete picture of the experiences in the end of life of people with PD and their family caregivers.

### Ethical issues

Sensitive one-to-one interviews will be performed with PD patients with minimized risk of any harm, distress or inconvenience to participants. Ethical approval was received from the ethics committee Arnhem-Nijmegen [number; 2016–2424] in February 2017. In order to minimize the burden of data collection during the multiple case study, questionnaires have a short completion time and the total duration of interviews will be limited to a maximum of 45 min. The interview will be interrupted if a PD patient has an ‘off-phase’ and will continue later on or scheduled another day. Interviews will be conducted by researchers who have experience in the care for advanced PD patients/family caregivers and qualitative research.

We expect that PD patients in our study may have cognitive deficits or develop dementia during the study. We expect most patients to be cognitively able to comprehend the aims, but we will evaluate participation if patients show signs of dementia and are unable to complete questionnaires or participate in interviews.

## Discussion

Current knowledge about palliative care in PD is scarce. This study is the first multifaceted and mixed-methods study that will provide valuable knowledge about the experiences of patients, family caregivers, bereaved family caregivers and professionals. The results can be used to directly improve patient care, and to improve policy decisions on PD palliative care. Furthermore, we will use the input from this study to inform a future intervention study in advanced PD.

The recruitment of professionals in studies can be problematic. A strength of this study is the presence of the national ParkinsonNet infrastructure within the Netherlands. [31, 32] Therefore, we have easier access to professionals who work regularly with PD patients’ in the end of life. ParkinsonNet give us the opportunity to readily find the various relevant professional disciplines. Furthermore, the infrastructure of ParkinsonNet will help us to find PD patients and bereaved family caregivers through contacts with professionals.

The multiple-case study design allows for an in-depth and longitudinal exploration of the palliative care trajectory of PD patients and family caregivers. The prospective design allows us to gain insight in needs and burden change over time. Identifying patients at the end of life will be a challenge. We use the “surprise question” but we do not know if this question is appropriate in PD. [12] By using the “surprise question” we might identify patients who still have a life expectancy longer than 12 months or those with a very short life expectancy of a couple of weeks. However, there is no evidence based instrument or point of access to identify the end of life. This motivated our choice for additional inclusion criteria next to the surprise question. We follow PD patients and their family caregivers for 12 months, assuming this is sufficient to also capture the terminal phase. A small sample of around 5–15 PD patients and their family caregivers will be included. This small sample size and heterogeneity of the PD population may lead to diversity in characteristics of PD patients and their family caregivers. This might lead to a broad insight in perspectives of the patients within our sample, which is a strong aspect in qualitative research. Therefore, we think that this study will gain more knowledge about the care for patients with advanced PD. This knowledge will be used for several purposes: to develop a new multidisciplinary guideline for professionals working with patients with PD; to create specific information materials (e.g. film, leaflet) for patients and caregivers; and to mutually inform and connect the existing networks of professional caregivers coming from Parkinson care and palliative care.
